# The role of muscle synergies and task constraints on upper limb motor impairment after stroke

**DOI:** 10.1007/s00221-024-06953-1

**Published:** 2025-01-08

**Authors:** Pablo Ortega-Auriol, Winston D. Byblow, April Xiaoge Ren, Thor Besier, Angus J. C. McMorland

**Affiliations:** 1 Department of Exercise Sciences, University of Auckland, Auckland, New Zealand; 2 Centre for Brain Research, University of Auckland, Auckland, New Zealand; 3 Auckland Bioengineering Institute, The University of Auckland, Auckland, New Zealand

**Keywords:** Muscle synergies, Impairment, Stroke, FuglmMaeyer, Upper limb, Motor control

## Abstract

This study explores the role of task constraints over muscle synergies expression in the context of upper limb motor impairment after stroke. We recruited nine chronic stroke survivors with upper limb impairments and fifteen healthy controls, who performed a series of tasks designed to evoke muscle synergies through various spatial explorations. These tasks included an isometric force task, a dynamic reaching task, the clinical Fugl-Meyer (FM) assessment, and a pinch task. Electromyographic data from 16 upper limb muscles were collected during each task, alongside intermuscular coherence (IMC) measurements during the pinch task to assess neuromuscular connectivity. The findings confirm that motor impairment is inversely related to the diversity of muscle synergies, with fewer synergies and more stereotypical synergy structures observed post-stroke. The study further reveals that the nature of motor tasks significantly affects the number of identifiable muscle synergies, with less constrained tasks revealing a broader array of synergies. These findings highlight the importance of carefully selecting motor tasks in the context of clinical research and assessments to understand a patient’s motor impairment, thus aiding in developing tailored rehabilitation strategies.

## Introduction

Stroke is a leading cause of adult disability and mortality (Feigin et al. 2014; GBD 2016 Stroke Collaborators 2019). Reduced upper limb function is the second most common motor impairment after a stroke (Mayo et al. 1999). Several factors contribute to motor impairment, such as atypical muscle co-activation (Roh et al. 2015), reduced coordination and selectivity (Cheung et al. 2009a), spasticity (Langhorne et al. 2009), reduced neural drive (Nielsen et al. 2008), and reduced functional connectivity (Kisiel-Sajewicz et al. 2011). Upper limb recovery tends to plateau by six months and depends on the integrity of the corticospinal tract (Byblow et al. 2015). Despite decades of research into motor impairment, the development of therapies to achieve a consistent motor recovery of the upper limb remains challenging (Langhorne et al. 2009; Stinear 2017).

Measuring how much function is lost and regained is essential to enhance outcomes following a stroke. Several clinical and statistical tools have been developed to characterise the motor substrate after a stroke. A valid and reliable clinical tool to evaluate motor impairment is the Fugl-Meyer (FM) assessment (Fugl-Meyer et al. 1975). The Fugl-Meyer assessment was developed as a quantitative measurement of complex motor behaviour (Gladstone et al. 2002). The upper limb motor domain contains 33 items, of which at least 27 items focus on the capability and range of the upper limb degrees of freedom. The FM assessment aims to measure motor recovery, and its items are derived from the description of natural motor recovery after a stroke (Twitchell 1951). The FM assessment quantifies the isolated and combined range of motion of shoulder, elbow, and wrist movements, thus assessing the capabilities of the upper limb to navigate volumes of space.

The study of muscle synergies has led to new insights into healthy motor control as well motor impairment due to neurological disease or injury. Muscle synergies are defined as stable spatiotemporal patterns of muscle activity that facilitate coordinated movement (McMorland et al. 2015). They have been proposed as a modular coordination strategy to generate complex movements. Muscle synergies are routinely expressed in human limb movements (Berger and d’Avella 2014; Clark et al. 2009; d’Avella et al. 2006; Ivanenko et al. 2004; Krishnamoorthy et al. 2003; Marchis et al. 2015; Muceli et al. 2010; Ortega-Auriol et al. 2018) across different tasks, natural motor behaviours and unique scenarios. Muscle synergies have also been used to characterise residual motor impairment after stroke (Cheung et al. 2009b; Irastorza-Landa et al. 2021; Maistrello et al. 2021; Safavynia et al. 2011). Both the structural and temporal components of synergies change with impairment. The structural component exhibits fewer modules after a stroke (Cheung et al. 2009a; Hesam-Shariati et al. 2017), which may be the result of the merging and fractioning of the original modules (Cheung et al. 2012) and stereotypical changes, principally involving the obligatory co-activation of muscles (Kisiel-Sajewicz et al. 2011). The validity of muscle synergy approaches in tracking recovery after a stroke remains a topic of continuing research (Gregory Hong et al. 2021).

Clinical assessments and muscle synergy analyses are employed to evaluate remaining muscle coordination patterns post-stroke, with their effectiveness influenced by the extent of neurological damage, patient-specific factors, and assessment techniques. The items in the FM assessment test the ability of the upper limb to utilise the full range of biomechanical degrees of freedom. While the FM assessment is sensitive to the expression of muscle synergies that drive motor impairment, additional methods have been proposed to quantify changes in muscle synergy structure (Cheung et al. 2009b; d’Avella et al. 2003). To accurately extract all the available synergies from the upper limb, the analyses require the exploration of large spatial volumes (Burkholder and van Antwerp 2013). In addition, given the naturally variable nature of EMG signals, identifying muscle synergies requires extensive exploration and multiple repetitions (Steele et al. 2013; Tresch et al. 2006). Altogether, there is alignment between the exploration characteristics of the FM scale and the requirements for accurate synergy extraction, meaning the FM assessment may provide a reliable set of tasks to extract muscle synergies.

As clinical and muscle synergy assessments offer insights into the dynamics of muscle coordination, functional connectivity emerges as a critical factor in comprehensively understanding mechanisms of motor function recovery. The synchronisation of oscillatory activity between two distant functional units of the neuromuscular system is captured in functional connectivity measures (Farmer 1998). Functional connectivity between muscles has been suggested as a possible mechanism to perform (Farmer 1998) and correct (Pizzamiglio et al. 2017) movements, suggesting a primal role in motor performance. Intermuscular coherence (IMC) is a linear measure of functional connectivity based on correlation in the frequency domain that quantifies the shared information between muscles across the frequency spectrum (Bastos and Schoffelen 2016). IMC has been proposed as a possible biomarker for motor impairment (Farmer 1998; Fisher et al. 2012; Zheng et al. 2018), being sensitive to the motor and sensory changes that occur after stroke (Grosse 2002; Zheng et al. 2018). Lower IMC levels in the alpha, beta, and gamma bands have been observed in the EMG signals obtained from stroke participants compared to healthy controls (Larsen et al. 2017; Mima Tatsuya et al. 2001). After stroke, IMC between synergistic muscles is also lower than healthy controls (Kisiel-Sajewicz et al. 2011), but correlations between IMC and motor performance have not been identified to date.

Intermuscular coherence and muscle synergies have emerged as complementary assessments to elucidate the neural mechanisms of motor control. Although some studies have explored how muscle synergies and coherence vary across different muscle groups (Danna-Dos Santos et al. 2010; Marchis et al. 2015), analysis linking these phenomena to specific motor tasks and their interaction remains less explored. Research has focused on constrained tasks that are either isometric or dynamic, yet few studies have incorporated clinical measurements like the Fugl-Meyer assessment, which intuitively may offer a more comprehensive paradigm for evaluating motor impairment after stroke. The present study specifically investigates the influence of motor task selection over the assessment of motor performance after a stroke. The tasks chosen—ranging from isometric force to dynamic reaching, along with the clinical Fugl-Meyer assessment and fine motor control tasks like the pinch task—were selected to cover a broad spectrum of motor assessments. These tasks are commonly used paradigms to assess motor performance after stroke, enabling a comprehensive evaluation of how specific task constraints influence motor control mechanisms and muscle synergy expression.

## Methods

Our study included nine chronic stroke survivors with upper limb impairment and fifteen neurologically healthy age-matched controls (Table 1). Stroke-affected participants were included if they had upper limb impairment resulting from a single stroke event at least six months before the study. Stroke-affected participants were excluded if they had no active range of shoulder motion or could not comprehend the task instructions. Stroke-affected participants’ residual motor function was characterised by the upper Fugl-Meyer assessment (Fugl-Meyer et al. 1975; Gladstone et al. 2002). The control participants were included if they did not report any neuromuscular disease or incapacitating pain over the last six months before data collection. All participants gave written informed consent before any procedure. The University of Auckland Human Participants Ethics Committee (ID #023798) approved all procedures.

### Protocol

**Fig. 1:**
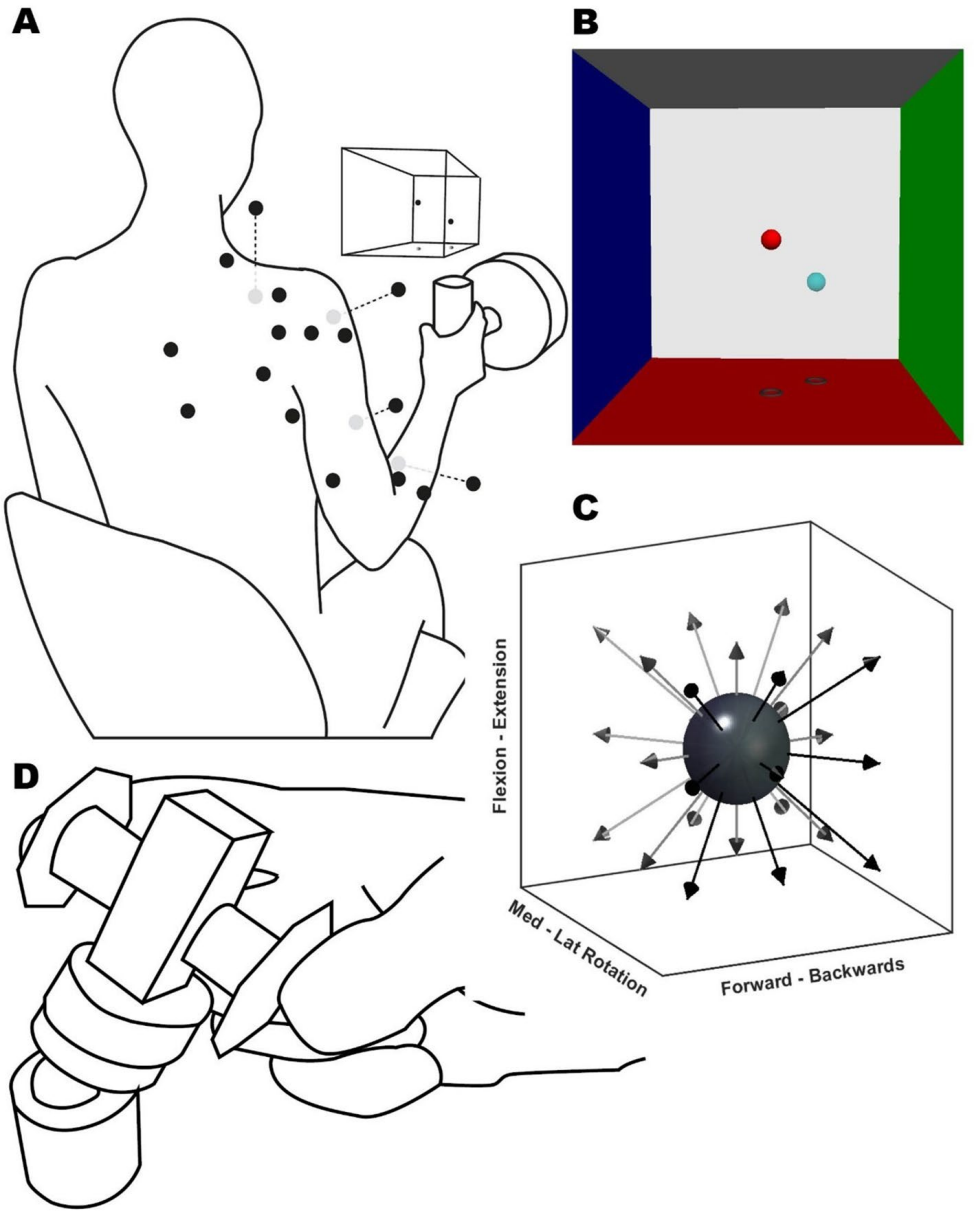
Experimental setup. (**A**) Illustration of the participant position and EMG sensor placement (black dots, grey dots are located ventrally), VRF view, and instrumented handle for the static task. A button replaced the handle for the dynamic task (not shown). (**B**) Screenshot of the VR feedback displayed on the screen, showing the target (light blue) and movable (red) spheres. Each VRF wall was located 100 N away from the centre. (**C**) Representation of target directions (arrows) for the static and dynamic task from a starting position (dark grey sphere). (**D**) Pinch task schematic, the participants exerted a concentric force onto a force transducer (centre cuboid) over a ball mount. The participant rested their forearms over the table; the height and pitch of the transducer were adjusted individually

The participants performed five tasks (Fig. 1). Three of these tasks aimed to elicit muscle synergies under spatial exploration volumes. The tasks consisted of producing (1) maximum voluntary force, (2) isometric directional force (static task), (3) a reaching movement (dynamic task), and (4) performing the Fugl-Meyer assessment (FM task). The fifth (5) task consisted of a finger pinch between the index and thumb fingers (pinch task). The stroke-affected group performed the tasks with their impaired limb, while the control group did it with their dominant UL, determined by the Edinburgh Inventory (Oldfield 1971). All participants performed the tasks in a single session and were trained on each task until they felt comfortable with their performance before data were collected.

**Table 1:** Demographic and clinical characteristics of stroke-affected and healthy participants

Stroke Affected Group							
ID	Age (Y)	Height (cm)	Weight (Kg)	Gender	Lesion Hemisphere	FM Score	Chronicity
A	69	178	100	M	R	17	137
B	74	184	85	M	L	19	117
C	56	172	71	F	L	58	31
D	77	177	76	M	R	61	24
E	77	180	80	M	L	48	39
F	70	185	91.5	M	R	39	8
G	61	183	100	M	L	44	14
H	80	180	70	M	R	36	67
I	78	175	92	M	R	52	56
**Average**	71.3	179.3	85.1			41.6	54.8
**SD**	7.7	4.1	10.9			14.7	42.7
**Healthy Participants**							
					**Dominance**		
A	63	168	67	F	R		
B	68	165	60	F	R		
C	73	169	55	M	R		
D	72	171	68	M	R		
E	69	180	90	M	R		
F	60	170	70	F	R		
G	76	152	51	F	R		
H	76	163	73	M	R		
I	74	181	79	M	R		
J	62	180	74	F	R		
K	68	160	69	M	L		
L	71	160	68	F	R		
M	61	159	73	M	R		
N	74	180	85	M	R		
O	54	175	80	M	R		
**Average**	69.1	168.4	70.1				
**SD**	5.4	8.9	10.2				

First, all participants were assessed on their maximal voluntary force (MVF). The MVF task consisted of three attempts at the maximum isometric force of the shoulder’s external rotation. The participants were seated with the shoulder at 0° and the elbow at 90° next to the trunk while grasping the handle. The shoulder’s external rotation is the weakest of all degrees of freedom. Force exertion in every direction of the following static task was normalised to 20% of the participant’s external rotation MVF (Roh et al. 2013).

During the static task (Fig. 1A), participants grasped the instrumented handle and exerted an isometric force over 26 virtually guided spatial directions. The handle position was in front of the biceps muscle belly at 40% of the arm’s length. The task consisted of two blocks of 26 different directions evenly distributed from the starting position (Fig. 1C). Custom Python-based virtual reality feedback (VRF) was used to orient the participant’s spatial force direction. The VRF consisted of two spheres (Fig. 1B), one fixed as the target and one movable as feedback. The movable sphere motion reflected the force transducer output in real-time. The movable sphere position relative to the centre point was the resultant vector of the force exerted at the handle, and the initial distance to the target was equal to 20% of the MVF (*±* 7 N). The goal of the task was to match the target sphere position with the movable sphere for four consecutive seconds. To inform the participant’s performance at the end of the trial, the spheres and box changed colours when the spheres matched and were held at the target level for four seconds.

During the dynamic task, subjects had to reach for and press a button mounted on a robotic arm (UR5, Universal Robots). The initial button position was the same as the handle in the static task, in front of the biceps muscle belly at 40% of the arm’s length. The participants’ hands’ starting position was with their palms resting on their thighs, directly underneath the starting position of the button. The robot moved in the same 26 directions as in the static task (Fig. 1C), again repeated for two blocks. For both dynamic and static tasks, trials were considered a miss if, after one minute, the participant could not match the target or press the button. Participants could self-pace both the static and dynamic tasks and take rests whenever needed to avoid fatigue.

The FM task consisted of participants from both groups executing the Fugl-Meyer motor assessment. The participants’ EMG activity was recorded only from the active movement items of the assessment. A researcher explained and demonstrated each item while the participant mirrored the movement. Once the participant understood the movement, five continuous repetitions of each item were recorded.

Finally, the pinch task consisted of an isometric pinch of a force transducer held between the index finger and thumb (Fig. 1.**D**). The goal of the task was to match a force-time trace displayed on a screen in front of them. The twenty trials consisted of an initial three seconds of rest, followed by a force-increasing ramp of two seconds, reaching a plateau of 6 N, then a hold period of eight seconds at 6 N (*±* 0.5 N), and finally back to rest in another two seconds. If the participant went out of the trace limits during the hold phase of the trial two times, the trial was discarded and repeated. In the original study design, we aimed to conduct an intermuscular analysis and a musculo-synergy coherence analysis during a static trial, as detailed in our previous publication (Ortega-Auriol et al. 2023). However, participants in the stroke group struggled with multiple repetitions of the static trial, which are essential for accurate coherence estimations. Consequently, we adapted our approach and measured intermuscular coherence (IMC) through the pinch task. While requiring a more complex analysis, this task enabled participants to perform sufficient repetitions, enhancing our data’s robustness.

### Devices

Forces during the static and dynamic tasks were recorded with an instrumented handle (Fig. 1) with a six-axis force-torque transducer (Omega160, ATI, Apex) at 1 kHz. Forces during the pinch task were recorded with a single-axis force transducer (MLP-100, Transducer Techniques) at 100 Hz mounted on a custom 3D printed platform.

Surface EMG signals were recorded with a Trigno System (Delsys Inc.) via a custom Python interface at 2 kHz. EMG was recorded from the participants’ dominant upper limb or paretic arm from the control and stroke-affected groups, respectively. For the static, active and FM tasks, we recorded the activity from 16 muscles or muscle portions: superior (ST), middle (MT) and inferior trapezius (IT), supraspinatus (Sup), infraspinatus (Inf), teres minor (TM), serratus anterior (SA), anterior (AD), middle (MD), and posterior deltoid (PD), pectoralis major (PM, clavicular fibres), long head of biceps brachii (Bic), long head of triceps brachii (Tr), brachioradialis (Brac), extensor carpi radialis (ECR), and flexor carpi radialis (FCR). Muscles were selected based on their relative contribution to the required tasks to accurately reconstruct all available synergies (Steele et al. 2013). During the pinch task, the hand’s first dorsal interosseous (FDI) and flexor pollicis brevis (FPB) were recorded with Trigno’s mini sensors (Delsys Inc.). Participants’ skin was prepared with a mild abrasive gel to decrease impedance, and all electrodes were positioned according to SENIAM and Cram’s guidelines (Hermens et al. 2000).

### Pre-processing

Synchronisation across devices was performed with a Python-based custom software (Dragonfly, Pittsburgh, 2017). Data analysis was performed in MATLAB 9.3 (MathWorks) using custom-made scripts and the FieldTrip toolbox (Oostenveld et al. 2011).

EMG data pre-processing was the same for the static, dynamic and FM tasks and similar to a previous study (Ortega-Auriol et al. 2018). In summary, EMGs were band-pass filtered (Butterworth, 2nd order, 10–400 Hz), demeaned, full-wave rectified, normalised to each muscle’s maximum activation across trials, converted to unit variance, low-pass filtered again (10 Hz) to calculate the envelope, trimmed to specific windows of interest, and finally rebinned into 100 data points.

Before rebinning, the trials of each task were trimmed to identify specific windows of activity. The static task trials were trimmed to the intermediate three seconds of the target match. Force signals were used to identify movement initiation (the point where the resultant force vector was greater than 5 N), and trials were ended after 4 s of matching between the VR spheres. Dynamic trials were trimmed from the start of the participant’s movement until touching the target. FM trials were trimmed to contain two repetitions of each movement item of the FM assessment. Pre-processed, trimmed trials’ EMG data were then concatenated into a single matrix to use as input for synergy extraction.

EMG data from the pinch tasks were used to calculate intermuscular coherence. EMG was first pre-processed by trimming the intermediate five seconds of the force hold period, band-pass filtered (5–100 Hz), demeaned and rectified using the Hilbert transform (Hilbert 1953). Signals were rectified to emphasise the grouping and timing of action potentials within the EMG signals as recommended for correlation and coherence analysis. Then, IMC was calculated between the EMGs of the FDI and APB muscles.

### Muscle synergies extraction

Muscle synergies were extracted independently from the concatenated EMG data from static, dynamic, and FM tasks. Non-negative matrix factorisation (NMF) (Lee and Seung 1999) was applied to each participant’s EMG data matrix for each task, resulting in a different set of synergies per task. NMF results can be modelled as, where D is the original data set, W synergy structure, C activation coefficients, and the error. NMF was implemented using the multiplicative rule (Berry et al. 2007). The final solution was the convergence of 20 consecutive iterations with a difference smaller than 0.01. The number of extracted synergies was iterated from one to the number of recorded muscles minus one. Then we used the variance accounted for (VAF) metric (Cheung et al. 2005) to determine the minimal significant number of synergies that accurately reconstructed the original data (Roh et al. 2013). The significance threshold was defined as VAF *≥* 90% (Cheung et al. 2005; Chvatal and Ting 2012; Kim et al. 2016; Ortega-Auriol et al. 2018; Roh et al. 2013).

### Synergies clustering

Once a significant number of synergies were extracted, we applied a cluster analysis to each set of synergies (from each task) and groups separately. Clustering allows us to group similar synergies across participants within each task and group. We used a k-medoids cluster analysis, with a cosine function as the distance metric between the members and centroids. The number of clusters was fixed to the maximum number of synergies in any single participant for each task and group. Membership assignment within a cluster was also constrained to avoid the repetition of two or more synergies from a single participant within a cluster. The repeated synergy within a cluster from any individual participant was reassigned to the next closest available (without any other synergy from the same participant). The reassignment process was iterated until no further repetitions were found. These methodological choices allowed NMF extraction to precede the clustering group distance metric for the initial assignment.

A second cluster analysis, now across tasks, was then applied to pair synergies across the tasks of each group. This second cluster analysis was applied to the pooled mean synergies from each cluster from the first analysis. The same restrictions regarding the number of identified clusters and membership repetition were applied until no further repeats were found. Finally, a third cluster (across groups) analysis was used to pair the synergies across the non-stroke and stroke groups. This third cluster analysis was applied to the pooled average synergies across tasks from the second cluster analysis. The same constraint criteria were used for the number of identified clusters and membership repetition.

### Inter muscular coherence

IMC was calculated for each participant from the pinch trials between the APB and FDI muscles. Pre-processed EMG signals from the pinch task were transformed into the frequency domain by applying a fast Fourier transform (FFT). A three-multitaper (spectral smoothing) FFT was applied between 5 and 100 Hz in bands with steps of 3 Hz, resulting in 32 frequency bins. A discrete prolate spheroidal sequence (dpss) was used to prevent leakage, maximising the energy in the main lobe of the window relative to the total power of the analysed segment (Ghil and Taricco 1997). IMC for each participant was calculated with Eq. 1.

$${C_{xy}}\left(f \right) = \,\frac{{{{\left| {{P_{xy}}\left(f \right)} \right|}^2}}}{{\left| {{P_{xx}}\left( f \right)} \right|{P_{yy}}\left(f \right)|}}$$

Once calculated, raw IMC values were normalised by applying a z-transformation (Baker et al. 2003; Reyes et al. 2017) with Eq. 2:

$$Z\, = \,\frac{{{\text{arctanh}}(\sqrt c)}}{{\sqrt {1/2N} }}$$

where c is the raw IMC value, and N is the number of tapers used in the IMC calculation. IMC was considered significant if its value surpassed a threshold. The threshold was derived from the surrogate time series of the original EMG data from the non-stroke group: after the original data were transformed into the frequency domain, the argument of the complex quantity (angle of the polar form) was independently shuffled. The shuffling was iterated 50 times across all trials, channels, and participants. Then, coherence was calculated as described previously. This procedure allows the conservation of the power spectrum original amplitude structure of the signal while only shifting the signal phase, uncorrelating the signals in the time and frequency domain (Faes et al. 2004; Marchis et al. 2015). Coherence threshold significance was established above the 90th percentile of the resultant by-chance coherence distribution. Finally, we calculated the IMC area under the curve (AUC) using a trapezoidal numerical integration approach and then calculated the mean IMC above the threshold. The mean area under the curve and mean IMC value were used as the participant’s pinch task-relevant outcomes.

**Statistical analysis**: We applied a mixed ANOVA to analyse differences in the number of synergies between groups (a between-subjects factor: stroke and non-stroke) and within three tasks (a within-subjects factor: static, dynamic, FM). Third, we used linear regression to quantify the relationship between the number of synergies and FM scores for each task. To analyse how similar muscle synergy clusters are within each group, we used a Wilcoxon test to examine the pooled dot product similarities across different muscle synergies. This statistical test allows us to determine whether synergies are consistently replicated within the stroke and non-stroke groups by comparing the magnitudes of dot products, which quantify the alignment between pairs of synergy vectors. Finally, to determine the relationship between IMC and other motor function characterisation methods, we used linear regression to quantify the relationship between the FM score and AUC, FM score and mean coherence above the threshold, number of synergies and AUC, and number of synergies and mean IMC.

## Results

All non-stroke participants were able to complete all the tasks. Within the stroke group, five of the nine participants completed the static task, six the pinch task, and all nine completed the dynamic and FM tasks.

**Fig. 2:**
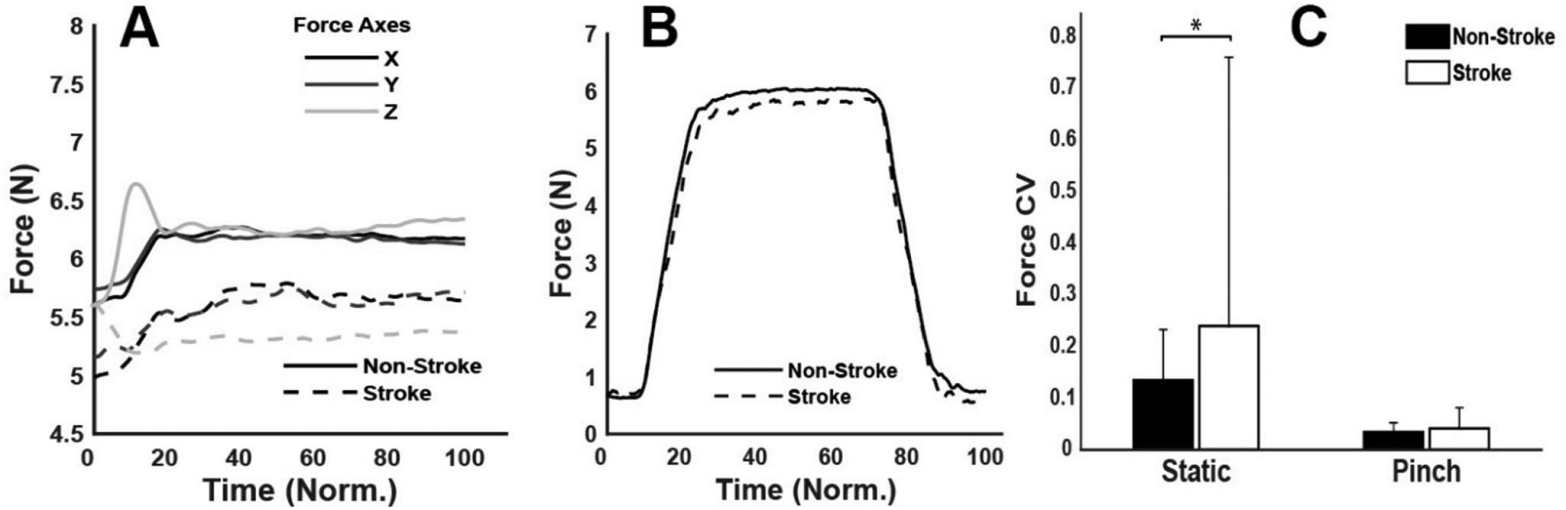
Average group force traces from the (**A**) static task and (**B**) pinch task, and comparison of force coefficient of variation (CV) between the stroke and non-stroke groups. Time is normalised (Norm) across the duration of the task

During the maximum voluntary force trial, the non-stroke participants exhibited a 27% higher force output [73.2 (14.8) N; mean (SD)] compared to the stroke participants [57.7 (22.6) N], though this difference was not statistically significant (*p* = 0.07), indicating a trend towards higher force generation by 27%. Over the static and pinch trials, despite the similarity in force profiles between both groups, as shown in Fig. 2, significant differences were observed in the force coefficient of variation (CV), indicating variability. For the static task, the non-stroke group demonstrated significantly lower variability [CV = 0.13] compared to the stroke-affected group [CV = 0.24], t(22) = -5.06, with a small effect size (d = 0.36) (Rosenthal 1986). Similarly, in the pinch task, the non-stroke participants showed lower variability [CV = 0.03] than the stroke group [CV = 0.04], t(22) = -1.97, *p* = 0.05, again indicating a small effect size (d = 0.29) as depicted in Fig. 2C.

**Fig. 3:**
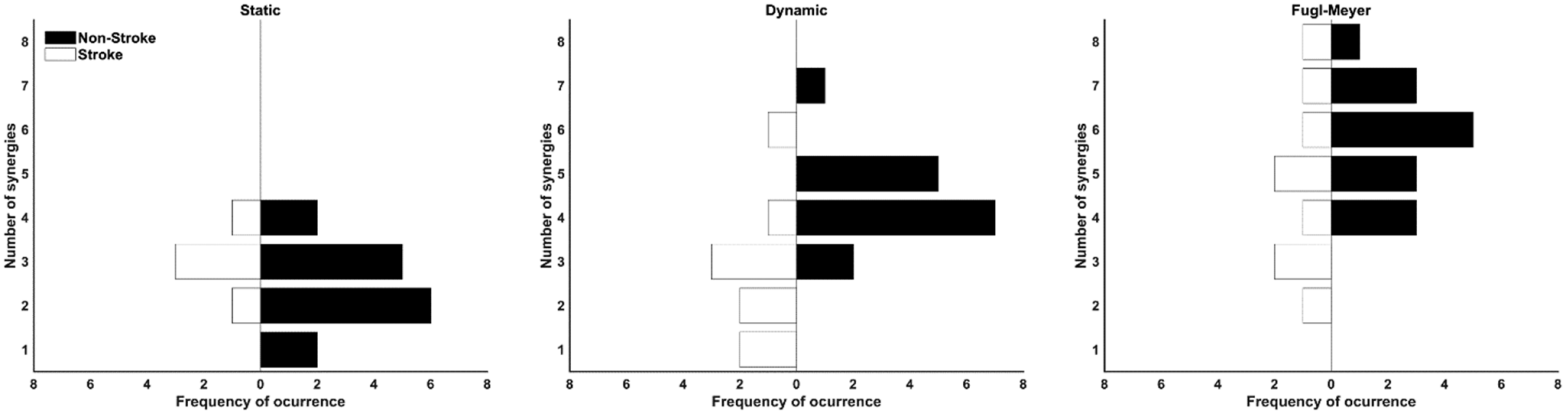
Frequency of occurrence of the number of extracted synergies for the stroke (white bottom bars) and non-stroke (top black bars) groups per task

Eight was the maximum number of extracted muscle synergies across tasks and groups (FM task for both groups) (Fig. 3). The non-stroke group presented more synergies than the stroke group (Table 2). The mixed ANOVA (Table 2) reported no interactions between tasks and groups. Similarly, there was a within-task effect where pairwise comparisons showed significant differences in the number of synergies found between the FM and other tasks (Table 2).

**Table 2:** Mean number of muscle synergies per task and group, ANOVA results indicating differences across tasks and groups, and Bonferroni-corrected pairwise comparisons of task performance

Synergies per Task and Group				
Group	Static	Dynamic	Fugl Meyer	
Non-stroke	2.5 (0.9)	4.4 (0.1)	6.7 (1.2)	
Stroke	3 (0.7)	2.8 (1.6)	4.8 (0.2)	
**ANOVA Results**				
**Model**	**DF**	**F**	**p**	**ES (η_p²)**
Task	2 (36)	33.7	0.0002	0.65
Group	1 (18)	1.7	*0.04*	0.62
Task * Group	2 (36)	2.4	0.1	0.12
**Pairwise Comparisons**				
**Model**	**Δ Difference**	**p**	**95% CI Bottom**	**Upper**
Static–Dynamic	-0.97	0.107	-2.09	0.16
Static–FM	-2.73	0.00005	-3.98	-1.49
Dynamic–FM	-1.77	0.002	-2.88	-0.65

**Fig. 4:**
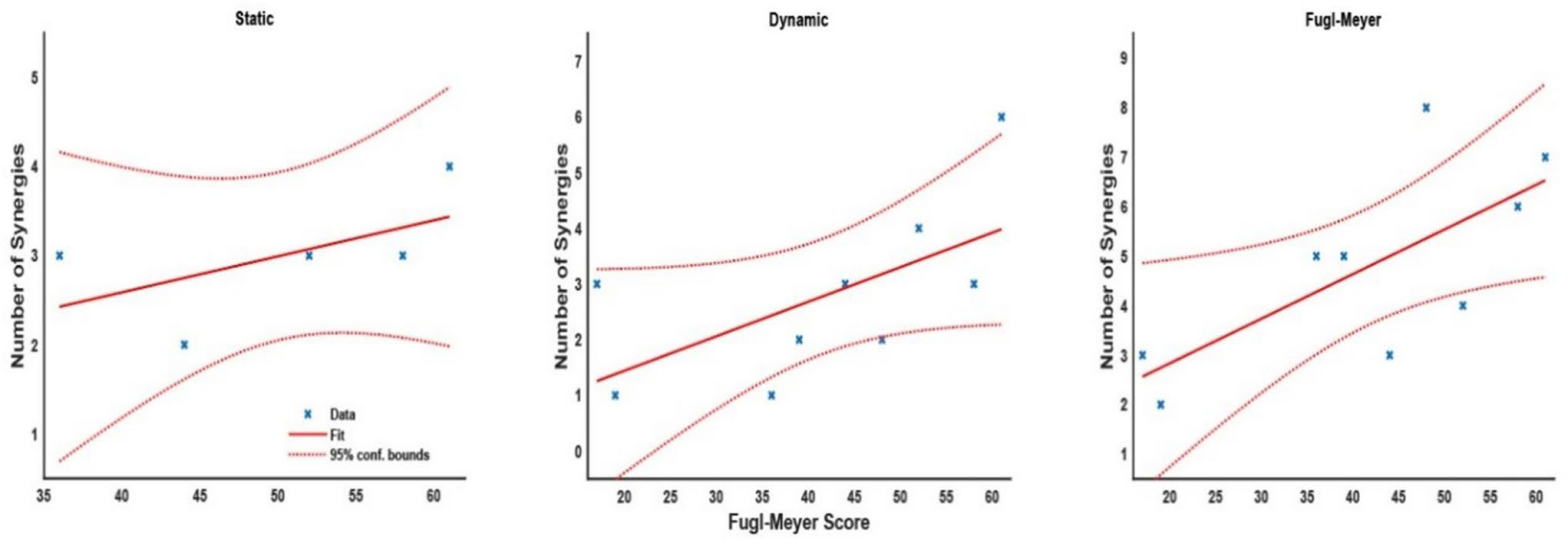
Scatter plot and linear regression fit of the FM score and the number of synergies for the stroke group in each task. Dashed lines show 95% CI for the regression fit

The number of extracted synergies represents the diversity of motor strategies: fewer synergies suggest a simpler motor pattern, capturing a large portion of the EMG data variance, whereas a greater number of synergies indicates a more complex motor strategy. The idea is nicely reflected by the statistical models, where the linear regression between the number of synergies and FM scores (Fig. 4) showed positive relationships across all tasks for the stroke group. In the static task, regression analysis suggested that the number of synergies slightly increased by 0.4 for every 10 points in the FM score (m = 0.04, intercept = 0.97, t = 1.25, *p* = 0.3) with a relatively low R^2^ = 0.34. In the dynamic task, synergies increased by 0.6 for every 10 points in the FM score (m = 0.06, intercept = 0.20, t = 2.09, *p* = 0.08) with a low R^2^ = 0.38. Finally, the FM task showed an increase of 0.9 synergies for a 10-point increase in the FM score (m = 0.09, intercept = 1.03, t_m_ = 2.66, p_m_ = 0.03) with a moderate R^2^ = 0.5.

**Fig. 5:**
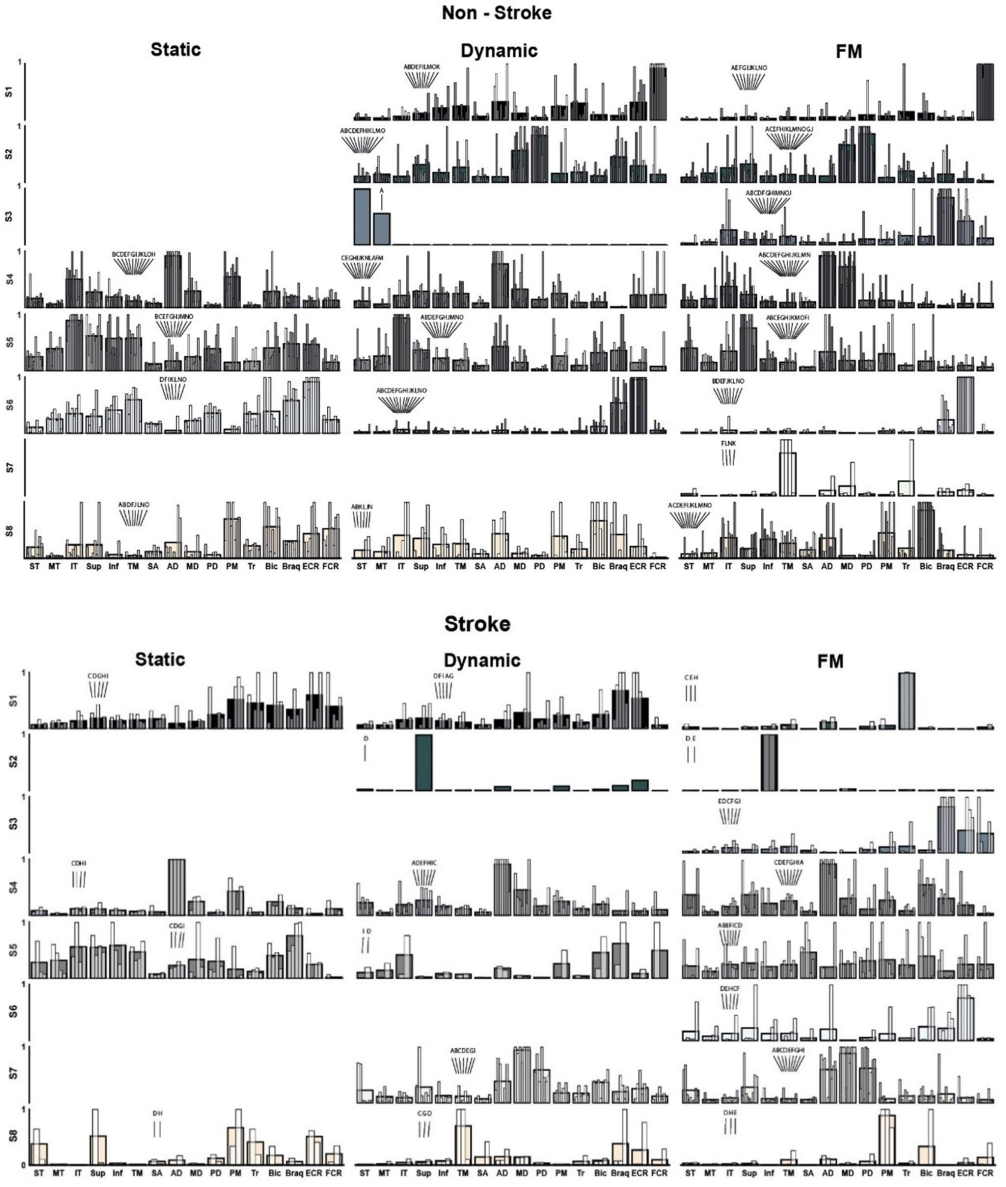
Individual (white overlaid bars) and clusters mean of normalised synergies (greyscale bars) for the non-stroke (top) and stroke groups (bottom). Eight synergy clusters (S1–S8) were grouped according to the maximum number of identified synergies across participants for each task and group. Three consecutive cluster analyses allowed the grouping of synergies across tasks and study groups. Letters above the white overlaid bars correspond to each participant ID, as in Table 1. Muscles are labelled in an abbreviated form: superior (ST), middle (MT) and inferior trapezius (IT), supraspinatus (Sup), infraspinatus (Inf), teres minor (TM), serratus anterior (SA), anterior (AD), middle (MD), and posterior deltoid (PD), pectoralis major (PM), long head of biceps brachii (Bic), long head of triceps brachii (Tr), brachioradialis (Brac), extensor carpi radialis (ECR), and flexor carpi radialis (FCR)

A different number of clusters was identified for each task and group; this number was constrained to the highest number of synergies extracted from any single participant for that task and group (Fig. 5). Four clusters were identified for the static task for both groups. Six and seven clusters were identified for the stroke and non-stroke groups for the dynamic task. Finally, eight clusters were extracted from the FM task for both groups.

The functional interpretation for each synergy can be complex, and the commonality of a synergy across participants must be considered given that some synergies (e.g. non-stroke, dynamic, S3 synergy) were present on a single or just a few participants. Overall:

- In the control group, Synergy S1 functions as a wrist flexor (FCR), elbow extensor (Tr), and shoulder internal rotation-flexion (AD, PM). For stroke survivors, S1 demonstrates co-contractions in upper limb muscles during static and dynamic tasks, indicating significant differences from non-stroke individuals. Notably, its presence in only two individuals during FM tasks suggests these stroke survivors might have retained more selective motor control, making S1 a potential target for rehabilitation strategies aimed at enhancing recovery and motor function.
- In the control group, Synergy S2 facilitates shoulder abduction and extension (MD, PD), along with elbow flexion (Brac), engaging primarily during dynamic movements. This synergy is rare among stroke survivors, where it manifests predominantly with just one muscle activation. This highlights a significant reduction in motor coordination complexity. S2 might be a critical marker in assessing and targeting recovery strategies in stroke rehabilitation.
- For Synergy S3 in the control group, the functionality is consistent across participants, primarily acting as an elbow and wrist stabiliser (Brac, FCR) during the FM task. This synergy is also present among stroke survivors, suggesting its retention post-stroke.
- In all tasks, Synergy S4 primarily acts as a shoulder abductor, engaging the same muscles (AD, MD) in both stroke and non-stroke groups. This commonality suggests a basic, preserved capability for shoulder control after a stroke.
- Synergy S5 involves external shoulder rotation (IT, Sup, TM, Inf) and elbow flexion (Bic, Brac). It primarily acts as a stabiliser for the scapula, well-defined in the control group. In stroke survivors, S5 is less consistently observed, especially during dynamic reaching tasks, suggesting it is part of the motor deficit.
- Synergy S6 functions as an elbow (Brac) and wrist (ECR) stabiliser in both control and stroke groups, with an added external shoulder rotation component (TM, Inf) in the static task. It is commonly observed across all tasks in the control group but is only present in the FM task for stroke survivors.
- Synergy S7, which is present in only four controls, functions primarily for shoulder external rotation (TM) and elbow extension (Tr). In stroke survivors, however, S7 exhibits a significant alteration, co-activating all three deltoid portions. This change is indicative of post-stroke motor control challenges and is observed in dynamic and FM tasks, suggesting its active role beyond isometric contractions.
- Synergy S8 consistently functions as a shoulder internal rotator–flexion (PM, AD) and elbow flexor (Bic) synergy across all tasks in control participants, maintaining a similar role. However, in stroke survivors, S8’s function appears altered, often isolating individual muscles with increased activation weights. While this synergy is prevalent in controls, it is present only in a few stroke survivors with high Fugl-Meyer scores.
- S8 served as shoulder internal rotator–flexion (PM, AD) and elbow flexor (Bic) synergy.

In the static task, S1 co-activated arm and forearm antagonist muscles (PM, Tr, Bic, Brac, ECR), whereas in the dynamic task, it showed a stabilising role akin to non-stroke S6, and during the FM task, it activated a single muscle (Tr). For the dynamic and FM tasks, S2 was characterised by single muscle activation (Sup). S3, only present in the FM task, was associated with forearm co-contraction (Brac, ECR, FCR). In both the static and dynamic tasks, S4 facilitated shoulder flexion and abduction (AD, MD) and incorporated a shoulder flexion component (Bic) in the FM task. S5 exhibited non-distinctive co-activation patterns across the upper limb muscles, while S6, present solely in the FM task and analogous to that in the non-stroke group, served as a stabiliser for the elbow (Brac) and wrist (ECR). S7 displayed an atypical synergy involving co-activation of all three deltoid portions, and S8 demonstrated a shoulder rotational synergy, with internal rotation (PM) and elevation (Sup) during the static task, external rotation (TM) for the dynamic task, and internal rotation (PM) in the FM task.

**Fig. 6:**
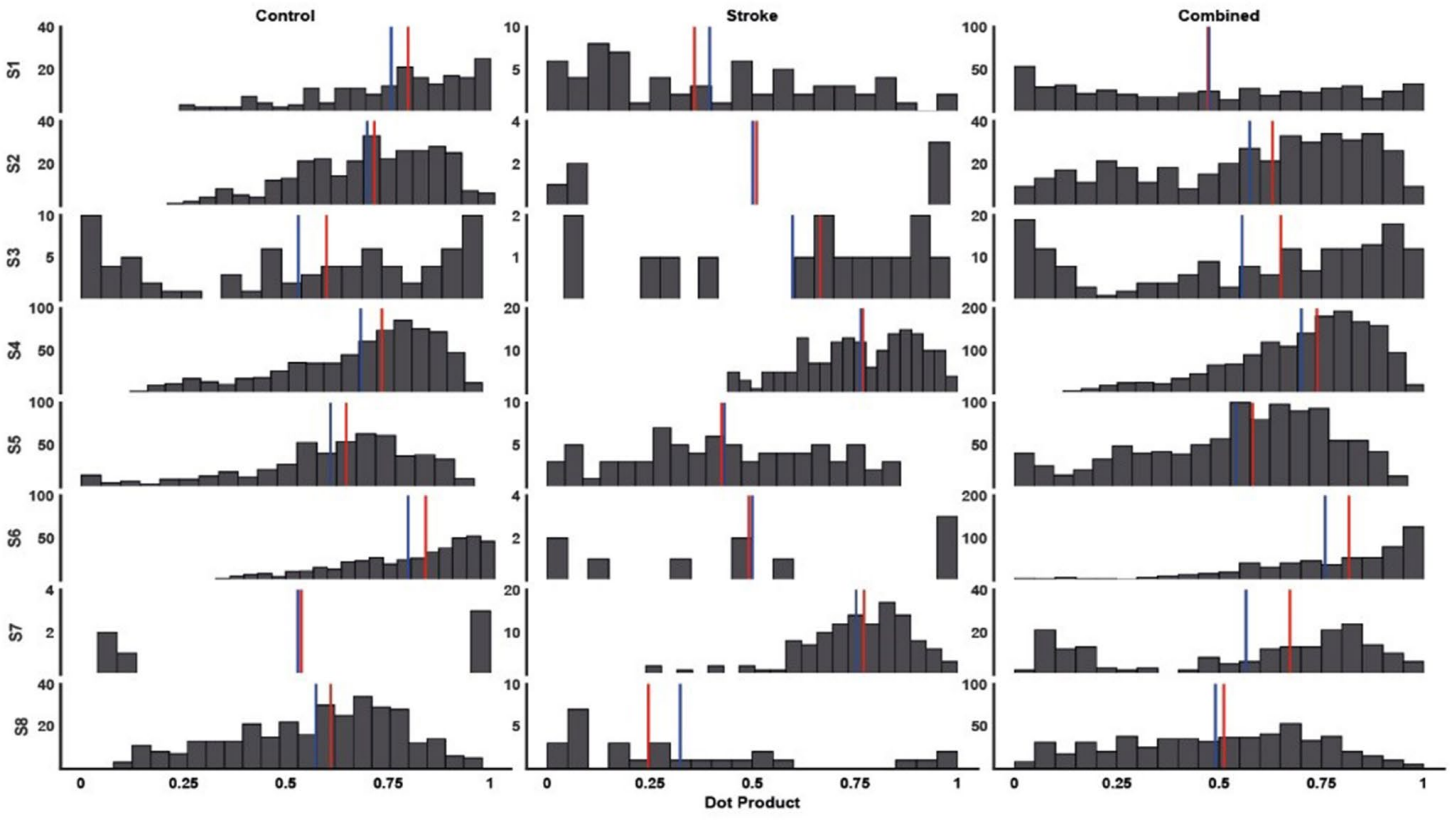
Histograms of cluster similarities (dot product), median value (vertical red line) and average (blue vertical line). Columns from left to right display the non-stroke, stroke and both groups combined similarities for each synergy (S1–S8). The Y-axes of each subplot represent the frequency of occurrence of a bin of the dot product (20 bins). The dot products for each group were calculated from every possible pairing between cluster members (S1– S8). Similarly, the combined dot products were calculated across all tasks and groups (S1– S8)

The intra-cluster dot product similarity was statistically higher in the non-stroke group [0.65 (0.1)] compared to the stroke group [0.53 (0.16)], with the combined group similarity at [0.58 (0.1)] (Fig. 6). A Wilcoxon test revealed statistically significant higher similarity in the non-stroke group’s synergies, though with a small effect size (z = 2.8, *p* = 0.001, *r* = 0.15).

**Fig. 7:**
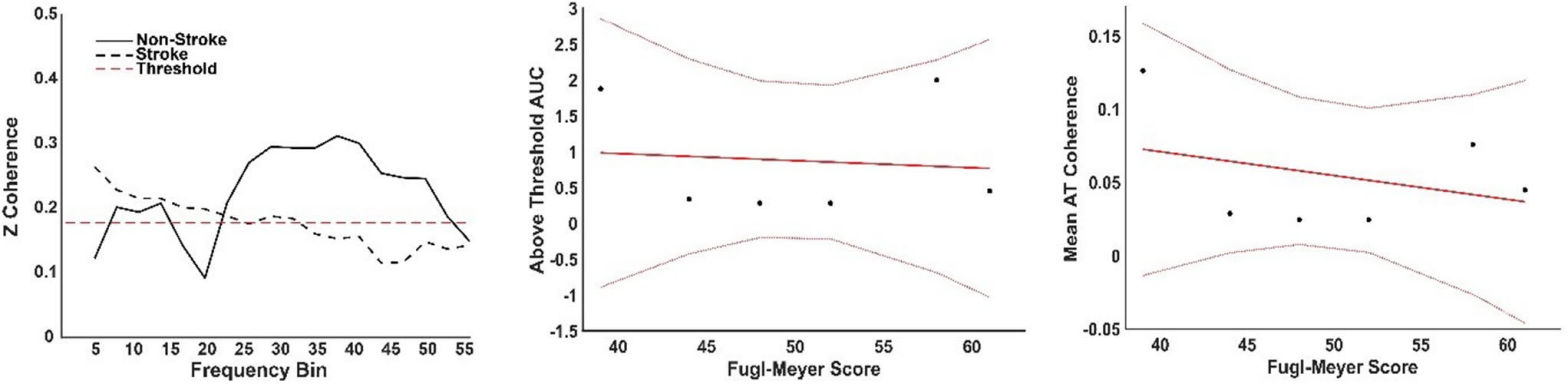
Analysis of Inter-Muscular Coherence (IMC). Left panel: IMC results for pinch tasks between the First Dorsal Interosseous (FDI) and Abductor Pollicis Brevis (APB) muscles, comparing non-stroke (solid line) and stroke groups (dashed line). Centre panel: Above-threshold (AT) area under the curve (AUC) for IMC, displaying data points and fitted model (solid red line) along with 95% confidence intervals (dotted lines). Right panel: Mean IMC of stroke participants plotted against Fugl-Meyer (FM) score, with the fitted model and 95% confidence intervals shown

**Fig. 8:**
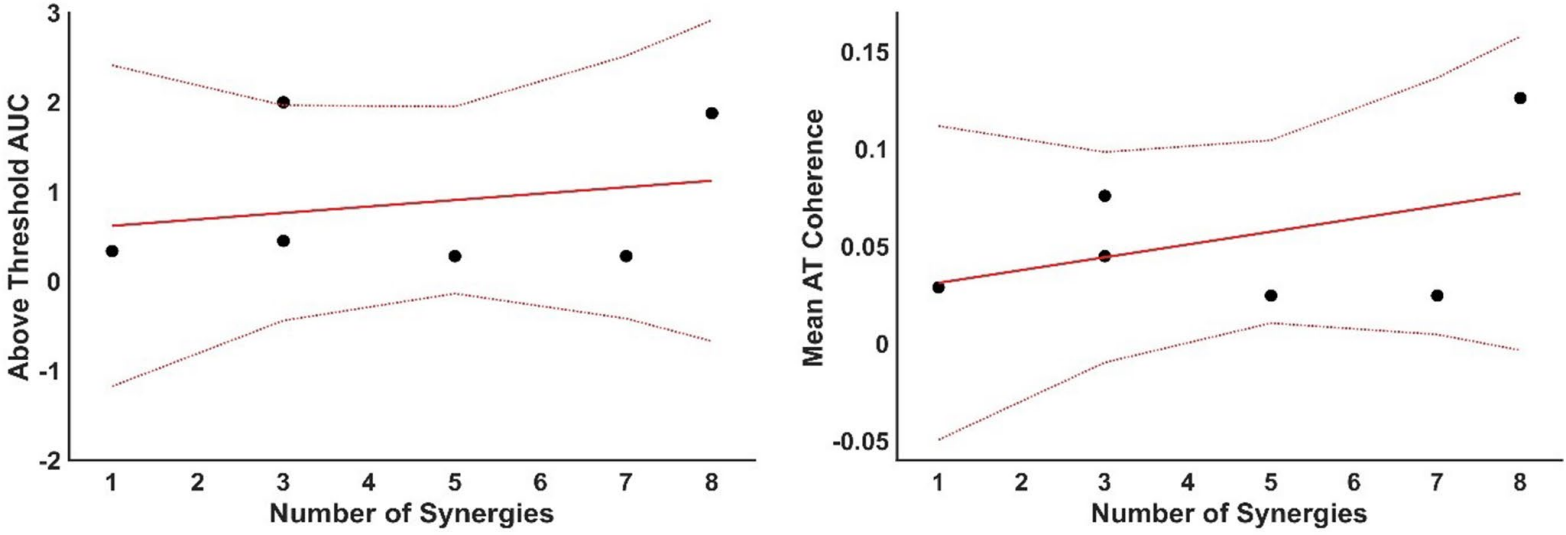
Relationship between muscle synergies and inter-muscular coherence. Left panel: Area under the curve (AUC) for Inter-Muscular Coherence (IMC) plotted against the number of muscle synergies (MSs) extracted from the Fugl-Meyer (FM) assessment task. Data points are shown alongside the fitted model (solid red line) and 95% confidence intervals (dotted lines). Right panel: Mean above-threshold z-coherence values plotted against the number of muscle synergies, with each data point representing an average from the FM task, accompanied by the fitted trend line and confidence intervals

Both groups had an above-threshold coherence level (Fig. 7. **A**), where the threshold was defined as the 95% percentile of a random distribution of coherence values. The non-stroke group, those able to perform the task (*N* = 6), showed two distinct peaks, the first including both alpha and lower beta bands and a second broader peak from beta until the gamma band. The total area under the curve (AUC = 1.7) for these two peaks was calculated from the mean intermuscular coherence (IMC) of the non-stroke (NS) group. The stroke group presented a low-frequency component (< 3 Hz), along with alpha and lower beta band components, with an AUC = 0.6 for the stroke group. The FM score and the AUC (Fig. 7B) were weakly negatively correlated (*R* = -0.12), suggesting a minimal association; the model explained less than 1% of the variance (R^2^ = 0.01) and was not significant (*p* = 0.085). Similarly, the FM score and the average Z-coherence above the threshold (Fig. 7C) were weakly negatively correlated (*R* = -0.34). The model explained 11.4% of the variance (R² = 0.114) and was not statistically significant (*p* = 0.51). No correlation was found between the IMC AUC and the number of muscle synergies (*R* = 0.01) or between the IMC average and the number of muscle synergies (*R* = -0.05) (Fig. 8).

## Discussion

The present study explored muscle synergies and intermuscular coherence across stroke-affected and healthy individuals. As expected, there were differences in motor performance between groups, with stroke-affected participants exhibiting fewer synergies and stereotypical abnormal synergy structures. This finding supports our hypothesis that motor impairment levels are inversely related to the diversity of muscle synergies. However, IMC did not show a direct relationship with motor impairment. IMC may be influenced by neuromuscular factors independent of muscle synergies and motor impairment. Task constraints were evident with less constrained tasks, such as those within the Fugl-Meyer assessment, eliciting a broader array of synergies. This finding highlights the impact of task variability on synergy expression.

After a stroke, force reduction is mainly explained by the subsequent corticospinal tract damage (Ward et al. 2006) and impaired cortical activation (Colebatch & Gandevia 1989; Ward 2004). As expected, we found a diminished force production of the stroke group during the static and pinch tasks. Furthermore, stroke-affected participants presented a higher force variability (Lodha et al. 2010) than non-stroke participants. Increased variability can be partially explained by motor changes after stroke, such as fluctuation of motor unit discharge rate (Moritz et al. 2005), degree of motor unit synchronisation (Taylor et al. 2003), and lower force production capacity (Sosnoff and Newell 2006). Nevertheless, given the small effect sizes, in a practical sense, stroke participants could perform similarly to non-stroke participants. All the stroke-affected participants who completed the static and pinch tasks showed a moderate-to-mild Fugl-Meyer score, highlighting the importance of overall motor impairment to accomplish tasks with a higher degree of accuracy.

In principle, more synergies reflect a higher capacity of motor complexity of individuals, able to recruit and combine modules from a broader repertoire. The present results showed a positive correlation between the number of synergies and motor function measured as the FM score, highlighting the relationship between motor impairment and the expression of independent synergies. The non-stroke group showed more synergies for the least constrained tasks (dynamic & FM tasks) than the stroke group. These results are consistent with previous reports of a stroke-affected population with fewer synergies across different tasks, either when comparing the more affected extremity with a control group or with the less affected limb (Clark et al. 2009; Pan et al. 2018; Runnalls et al. 2019). Different numbers of synergies can also be found during the different stages of motor recovery after a stroke. Early stages show fewer synergies, while in the later stages of recovery from a mild or moderate impairment, participants can exhibit similar synergies and comparable motor function to non-stroke participants (Pan et al. 2018). This evolution in the number of muscle synergies throughout recovery may be related to neural adaptation at cortical levels (Yao et al. 2009). Even when the number of synergies is not reduced after a stroke, studies have found stereotypical changes in the structure of the synergies, resulting in higher co-activation levels and less selective movement control (Cheung et al. 2009a, 2012; Roh et al. 2015).

The number of synergies varied across tasks for both groups, with more synergies present in the FM task, which is least constrained. At the same time, the identified synergies of the non-stroke group were consistent across tasks with only a few exceptions (i.e. dynamic S3). The conservation of muscle synergies across tasks suggests the recruitment of only a subset of the available synergies by the more constrained tasks. In the stroke group, the conservation of synergies across tasks is also observed. Some synergies, e.g. S8, were only expressed by a few participants. Consequently, those stroke-affected participants who exhibited a higher number of synergies, indicating more complex motor patterns, were those with higher FM scores (e.g., participants C and D), supporting a relationship between recovery from motor impairment and developing more complex synergy patterns. The finding of fewer synergies with more constrained tasks aligns with previous findings (Nazarpour et al. 2012) (Burkholder and van Antwerp 2013).

The present study demonstrates that task constraints and variability influence the identification of muscle synergies, with dynamic least-constrained tasks revealing a higher number of synergies than isometric ones due to their more complex motor actions and resultant more variable EMG signals. This EMG variability suggests that dynamic tasks lead to identifying more synergies, a concept supported by the impact of EMG filtering over the number of synergies (Hart and Giszter 2004; Santuz et al. 2017). Similarly, strategic data selection might refine synergy analysis by focusing on essential EMG segments (Ghislieri et al. 2018). This highlights that both the nature of the task and the methods used to process signals are crucial for understanding muscle coordination. Nevertheless, a consistent choice of methods across tasks in this study highlights that the observed differences are inherently linked to task characteristics, emphasising the importance of task selection in the assessments of muscle synergy.

Despite the apparent relationship between the number of synergies and motor impairment (Cheung et al. 2012), no previous studies have extracted muscle synergies during the FM-UE assessment. Synergies identified using constrained tasks may reveal only a subset of the entire repertoire of synergies. Other approaches have compared the usefulness of synergies and the FM score to assess specific tasks such as gait (Barroso et al. 2017; Bowden et al. 2010). These studies found a correlation between the number of synergies and gait parameters but not the FM-LE score, reflecting a potential independence between the FM-LE and more direct measures of gait after stroke (Barroso et al. 2017; Bowden et al. 2010). The intention of the FM is to elicit a broader repertoire of motor behaviours, providing a better insight into the available motor substrate of a participant than more constrained tasks such as gait. Our findings highlight the importance of considering volume exploration as a key parameter to capture the complete variability involved in human movement production.

Changes within the synergy structure are common after stroke. Alternative approaches have been proposed to explain these changes: the presence of conservation, fractionation, or merging of synergies (Cheung et al. 2012; Clark et al. 2009) and stereotypical changes in synergies’ structure (Roh et al. 2013, 2015), and these two theories are not mutually exclusive. In the present study, we observed stereotypical changes after stroke, particularly the co-activation of the three different portions of the deltoid muscle. In addition, the synergy with deltoid co-activation (stroke S7) was present in seven participants of the stroke group. Our findings support stereotypical deltoid recruitment changes after stroke (Roh et al. 2013, 2015). Although not described, the exact stereotypical change is present in other studies (Cheung et al. 2012; Runnalls et al. 2019; Scano et al. 2017; Tropea et al. 2013). These stereotypical changes in the shoulder abductors have even been described outside the muscle synergy framework (Dewald et al. 1995; McCrea et al. 2005). The mechanism that leads to this commonly observed change after stroke is worthy of further investigation.

IMC and muscle synergies can be considered complementary assessments to describe motor impairment. However, few studies have compared quantitative descriptions of muscle synergies and IMC across various muscles (Danna-Dos Santos et al. 2010; Marchis et al. 2015). We previously described that in healthy young adults, higher-weight muscles within a single synergy also display a higher IMC level, suggesting IMC may contribute to the emergence of synergies (Ortega-Auriol et al. 2023). Thus, similarly to muscle synergies, IMC may correlate with an overall motor performance estimate as the FM score. During the pinch task, we found higher IMC levels for healthy control participants within the alpha, beta and gamma bands than stroke participants. These results align with previous findings at the subacute stage of stroke (Larsen et al. 2017). The present results extend these findings to the chronic stroke population. Similarly, lower beta-gamma band IMC in the stroke group may reflect a partial loss of corticospinal input to alpha motor neurons directly or indirectly via propriospinal circuits (Zaaimi et al. 2012). Although the present findings did not show any relationship between IMC, the number of synergies or motor impairment patient data, low IMC has been reported after stroke (Kisiel-Sajewicz et al. 2011), along with stereotypical changes in the synergies structure (Cheung et al. 2009a; Israely et al. 2018; Roh et al. 2013, 2015). The lack of association between IMC and motor impairment suggests a low sensitivity of IMC as a biomarker for motor impairment.

Muscle synergy analysis in this study highlights distinct neuromuscular patterns that could inform targeted rehabilitation strategies for stroke survivors. For instance, Synergies S1 and S2, which show clear differences between stroke survivors and control participants, point to potential areas for enhancing motor performance. Additionally, the presence of preserved synergies across dynamic tasks suggests foundational motor capabilities that rehabilitation programs could aim to maintain and enhance. Incorporating dynamic broad tasks into rehabilitation could effectively challenge the motor system, potentially leading to better functional outcomes. Future research should aim to integrate muscle synergy analysis with task-specific rehabilitation approaches, providing a detailed understanding of motor function restoration and facilitating the design of more effective therapy regimens. This approach highlights the importance of task selection in clinical research and rehabilitation, where the complexity and nature of tasks can significantly influence the recruited synergies and the generalizability of research findings.

This study is constrained by the small sample size, potentially limiting the generalisability of the findings across the broader stroke population. Additionally, the functional requirements of the static task unintentionally restrict participation. While all participants performed the FM task, only those with higher FM scores could execute the static task, leading to a skewed representation towards less impaired individuals. This aspect highlights the challenge of task selection in stroke research, where task difficulty can exclude severely impaired individuals and potentially limit the applicability of findings across the full spectrum of stroke-related disabilities. Despite these limitations, the consistency observed within our sample suggests potential underlying patterns that merit further investigation. Future studies should aim to incorporate tasks that accommodate a wider range of functional abilities to ensure a more representative sample of the stroke population.

The present findings confirm previous observations that upper limb motor impairment levels are inversely related to the diversity of muscle synergies. Fewer synergies and stereotypical changes in synergy structure are evident after stroke. In the present study, task constraints significantly influenced the number of identified muscle synergies, such that less constrained tasks elicited a broader array of synergies. These results highlight the importance of task selection in clinical assessments and suggest that broader, less constrained tasks may provide deeper insights into a patient’s motor capabilities and recovery potential.

## Data Availability

Anonymised data or results can be available from the corresponding author upon reasonable request.

## References

[CR1] Baker SN, Pinches EM, Lemon RN (2003) Synchronization in Monkey Motor Cortex during a Precision grip Task. II. Effect of Oscillatory Activity on Corticospinal output. J Neurophysiol 89(4):1941–1953. 10.1152/jn.00832.2002 12686573 10.1152/jn.00832.2002

[CR2] Barroso FO, Torricelli D, Molina-Rueda F, Alguacil-Diego IM, Cano-de-la-Cuerda R, Santos C, Moreno JC, Miangolarra-Page JC, Pons JL (2017) Combining muscle synergies and biomechanical analysis to assess gait in stroke patients. J Biomech 63:98–103. 10.1016/j.jbiomech.2017.08.006 28882330 10.1016/j.jbiomech.2017.08.006

[CR3] Bastos AM, Schoffelen J-M (2016) A Tutorial Review of Functional Connectivity Analysis methods and their interpretational pitfalls. Front Syst Neurosci 9. 10.3389/fnsys.2015.00175 10.3389/fnsys.2015.00175 PMC4705224 26778976

[CR4] Berger DJ, d’Avella A (2014) Effective force control by muscle synergies. *Frontiers in Computational Neuroscience*, *8*. 10.3389/fncom.2014.00046 10.3389/fncom.2014.00046 PMC4029017 24860489

[CR5] Berry MW, Browne M, Langville AN, Pauca VP, Plemmons RJ (2007) Algorithms and applications for approximate nonnegative matrix factorization. Comput Stat Data Anal 52(1):155–173. 10.1016/j.csda.2006.11.006

[CR6] Boonstra TW, Breakspear M (2011) Neural mechanisms of intermuscular coherence: implications for the rectification of surface electromyography. J Neurophysiol 107(3):796–807. 10.1152/jn.00066.2011 22072508 10.1152/jn.00066.2011

[CR7] Bowden MG, Clark DJ, Kautz SA (2010) Evaluation of abnormal synergy patterns Poststroke: relationship of the Fugl-Meyer Assessment to Hemiparetic Locomotion. Neurorehabilit Neural Repair 24(4):328–337. 10.1177/1545968309343215 10.1177/1545968309343215 PMC4434590 19794132

[CR8] Burkholder TJ, van Antwerp KW (2013) Practical limits on muscle synergy identification by non-negative matrix factorization in systems with mechanical constraints. Med Biol Eng Comput 51(1):187–196. 10.1007/s11517-012-0983-8 23124815 10.1007/s11517-012-0983-8 PMC3582774

[CR9] Byblow WD, Stinear CM, Barber PA, Petoe MA, Ackerley SJ (2015) Proportional recovery after stroke depends on corticomotor integrity. Ann Neurol 78(6):848–859. 10.1002/ana.24472 26150318 10.1002/ana.24472

[CR10] Cheung VCK, d’Avella A, Tresch MC, Bizzi E (2005) Central and sensory contributions to the activation and organization of muscle synergies during natural motor behaviors. J Neuroscience: Official J Soc Neurosci 25(27):6419–6434. 10.1523/JNEUROSCI.4904-04.2005 10.1523/JNEUROSCI.4904-04.2005 PMC6725265 16000633

[CR11] Cheung VCK, Piron L, Agostini M, Silvoni S, Turolla A, Bizzi E (2009a) Stability of muscle synergies for voluntary actions after cortical stroke in humans. Proc Natl Acad Sci USA 106(46):19563–19568. 10.1073/pnas.0910114106 19880747 10.1073/pnas.0910114106 PMC2780765

[CR12] Cheung VCK, Piron L, Agostini M, Silvoni S, Turolla A, Bizzi E (2009b) Stability of muscle synergies for voluntary actions after cortical stroke in humans. *Proceedings of the National Academy of Sciences*, *106*(46), 19563–19568. 10.1073/pnas.0910114106 10.1073/pnas.0910114106 PMC2780765 19880747

[CR13] Cheung VCK, Turolla A, Agostini M, Silvoni S, Bennis C, Kasi P, Paganoni S, Bonato P, Bizzi E (2012) Muscle synergy patterns as physiological markers of motor cortical damage. Proc Natl Acad Sci 109(36):14652–14656. 10.1073/pnas.1212056109 22908288 10.1073/pnas.1212056109 PMC3437897

[CR14] Chvatal SA, Ting LH (2012) Voluntary and Reactive Recruitment of Locomotor Muscle Synergies during perturbed walking. J Neurosci 32(35):12237–12250. 10.1523/JNEUROSCI.6344-11.2012 22933805 10.1523/JNEUROSCI.6344-11.2012 PMC3465667

[CR15] Clark DJ, Ting LH, Zajac FE, Neptune RR, Kautz SA (2009) Merging of Healthy Motor Modules predicts reduced locomotor performance and muscle coordination complexity Post-stroke. J Neurophysiol 103(2):844–857. 10.1152/jn.00825.2009 20007501 10.1152/jn.00825.2009 PMC2822696

[CR16] Colebatch JG, Gandevia SC, DISTRIBUTION OF MUSCULAR WEAKNESS IN UPPER MOTOR NEURON LESIONS AFFECTING THE ARM (1989) Brain 112(3):749–763. 10.1093/brain/112.3.749. THE 10.1093/brain/112.3.749 2731028

[CR18] d’Avella A, Saltiel P, Bizzi E (2003) Combinations of muscle synergies in the construction of a natural motor behavior. Nat Neurosci 6(3):300–308. 10.1038/nn1010 12563264 10.1038/nn1010

[CR17] d’Avella A, Portone A, Fernandez L, Lacquaniti F (2006) Control of fast-reaching movements by muscle synergy combinations. J Neuroscience: Official J Soc Neurosci 26(30):7791–7810. 10.1523/JNEUROSCI.0830-06.2006 10.1523/JNEUROSCI.0830-06.2006 PMC6674215 16870725

[CR19] Danna-Dos Santos A, Poston B, Jesunathadas M, Bobich LR, Hamm TM, Santello M (2010) Influence of Fatigue on Hand Muscle Coordination and EMG-EMG coherence during three-digit grasping. J Neurophysiol 104(6):3576–3587. 10.1152/jn.00583.2010 20926609 10.1152/jn.00583.2010 PMC3007653

[CR20] Dewald JPA, Pope PS, Given JD, Buchanan TS, Rymer WZ (1995) Abnormal muscle coactivation patterns during isometric torque generation at the elbow and shoulder in hemiparetic subjects. Brain 118(2):495–510. 10.1093/brain/118.2.495 7735890 10.1093/brain/118.2.495

[CR21] Faes L, Pinna GD, Porta A, Maestri R, Nollo G (2004) Surrogate data analysis for assessing the significance of the coherence function. IEEE Trans Biomed Eng 51(7):1156–1166. 10.1109/TBME.2004.827271 15248532 10.1109/TBME.2004.827271

[CR22] Farina D, Negro F, Jiang N (2013) Identification of common synaptic inputs to motor neurons from the rectified electromyogram. J Physiol 591(10):2403–2418. 10.1113/jphysiol.2012.246082 23507877 10.1113/jphysiol.2012.246082 PMC3678033

[CR23] Farmer SF (1998) Rhythmicity, synchronization and binding in human and primate motor systems. J Physiol 509(Pt 1):3–14. 10.1111/j.1469-7793.1998.003bo.x 9547376 10.1111/j.1469-7793.1998.003bo.x PMC2230956

[CR24] Feigin VL, Forouzanfar MH, Krishnamurthi R, Mensah GA, Connor M, Bennett DA, Moran AE, Sacco RL, Anderson L, Truelsen T (2014) & others. Global and regional burden of stroke during 1990–2010: Findings from the Global Burden of Disease Study 2010. *The Lancet*, *383*(9913), 245–255 10.1016/s0140-6736(13)61953-4 PMC4181600 24449944

[CR25] Fisher KM, Zaaimi B, Williams TL, Baker SN, Baker MR (2012) Beta-band intermuscular coherence: a novel biomarker of upper motor neuron dysfunction in motor neuron disease. Brain 135(9):2849–2864. 10.1093/brain/aws150 22734124 10.1093/brain/aws150 PMC3437020

[CR26] Fugl-Meyer AR, Jääskö L, Leyman I, Olsson S, Steglind S (1975) The post-stroke hemiplegic patient. 1. A method for evaluation of physical performance. Scand J Rehabil Med 7(1):13–31 1135616

[CR27] GBD 2016 Stroke Collaborators (2019) Global, regional, and national burden of stroke, 1990–2016: a systematic analysis for the global burden of Disease Study 2016. Lancet Neurol 18(5):439–458. 10.1016/S1474-4422(19)30034-1 30871944 10.1016/S1474-4422(19)30034-1 PMC6494974

[CR28] Ghil M, Taricco C (1997) Advanced spectral-analysis methods. In *Past and Present Variability of the Solar-Terrestrial System: Measurement, Data Analysis and Theoretical Models* (pp. 137–159). IOS Press. 10.3254/978-1-61499-218-9-137

[CR29] Ghislieri M, Agostini V, Knaflitz M (2018) The Effect of Signal-to-Noise Ratio on Muscle Synergy Extraction. *2018 IEEE Life Sciences Conference (LSC)*, 227–230. 10.1109/LSC.2018.8572075

[CR30] Gladstone DJ, Danells CJ, Black SE (2002) The Fugl-Meyer Assessment of Motor Recovery after Stroke: a critical review of its Measurement Properties. Neurorehabilit Neural Repair 16(3):232–240. 10.1177/154596802401105171 10.1177/154596802401105171 12234086

[CR31] Gregory Hong YN, Ballekere AN, Fregly BJ, Roh J (2021) Are muscle synergies useful for Stroke Rehabilitation? Curr Opin Biomedical Eng 100315. 10.1016/j.cobme.2021.100315

[CR32] Grosse (2002) EEG–EMG, MEG–EMG and EMG–EMG frequency analysis: physiological principles and clinical applications. Clin Neurophysiol 113(10):1523–1531. 10.1016/S1388-2457(02)00223-7 12350427 10.1016/s1388-2457(02)00223-7

[CR33] Hart CB, Giszter SF (2004) Modular Premotor drives and Unit bursts as primitives for Frog Motor behaviors. J Neurosci 24(22):5269–5282. 10.1523/JNEUROSCI.5626-03.2004 15175397 10.1523/JNEUROSCI.5626-03.2004 PMC6729191

[CR82] Hermens HJ, Freriks B, Disselhorst-Klug C, & Rau G (2000) Development of recommendations for SEMG sensors and sensor placement procedures. J Electromyogr Kinesiol 10(5):361–374. 10.1016/S1050-6411(00)00027-4. https://www.sciencedirect.com/science/article/pii/S1050641100000274 10.1016/s1050-6411(00)00027-4 11018445

[CR34] Hesam-Shariati N, Trinh T, Thompson-Butel AG, Shiner CT, McNulty PA (2017) A longitudinal Electromyography Study of Complex movements in Poststroke Therapy. 2: changes in coordinated muscle activation. Front Neurol 8. 10.3389/fneur.2017.00277 10.3389/fneur.2017.00277 PMC5517410 28775705

[CR35] Hilbert D (1953) Grundzuege einer allgemeinen Theorie der linearen Integralgleichungen, Chelsea Pub. *Co., New York*

[CR36] Irastorza-Landa N, García-Cossio E, Sarasola-Sanz A, Brötz D, Birbaumer N, Ramos-Murguialday A (2021) Functional synergy recruitment index as a reliable biomarker of motor function and recovery in chronic stroke patients. J Neural Eng 18(4):046061. 10.1088/1741-2552/abe244 10.1088/1741-2552/abe244 33530072

[CR37] Israely S, Leisman G, Carmeli E (2018) Neuromuscular synergies in motor control in normal and poststroke individuals. Rev Neurosci 0(0). 10.1515/revneuro-2017-0058 10.1515/revneuro-2017-0058 29397390

[CR38] Ivanenko YP, Poppele RE, Lacquaniti F (2004) Five basic muscle activation patterns account for muscle activity during human locomotion. J Physiol 556(1):267–282. 10.1113/jphysiol.2003.057174 14724214 10.1113/jphysiol.2003.057174 PMC1664897

[CR39] Kim Y, Bulea TC, Damiano DL (2016) Novel methods to Enhance Precision and Reliability in muscle synergy identification during walking. Front Hum Neurosci 10. 10.3389/fnhum.2016.00455 10.3389/fnhum.2016.00455 PMC5023666 27695403

[CR40] Kisiel-Sajewicz K, Fang Y, Hrovat K, Yue GH, Siemionow V, Sun C-K, Jaskólska A, Jaskólski A, Sahgal V, Daly JJ (2011) Weakening of Synergist Muscle Coupling during Reaching Movement in Stroke patients. Neurorehabilit Neural Repair 25(4):359–368. 10.1177/1545968310388665 10.1177/1545968310388665 21343527

[CR41] Krishnamoorthy V, Goodman S, Zatsiorsky V, Latash ML (2003) Muscle synergies during shifts of the center of pressure by standing persons: identification of muscle modes. Biol Cybern 89(2):152–161. 10.1007/s00422-003-0419-5 12905043 10.1007/s00422-003-0419-5

[CR42] Langhorne P, Coupar F, Pollock A (2009) Motor recovery after stroke: a systematic review. Lancet Neurol 8(8):741–754. 10.1016/S1474-4422(09)70150-4 19608100 10.1016/S1474-4422(09)70150-4

[CR43] Larsen LH, Zibrandtsen IC, Wienecke T, Kjaer TW, Christensen MS, Nielsen JB, Langberg H (2017) Corticomuscular coherence in the acute and subacute phase after stroke. Clin Neurophysiol 128(11):2217–2226. 10.1016/j.clinph.2017.08.033 28987993 10.1016/j.clinph.2017.08.033

[CR44] Lee DD, Seung HS (1999) Learning the parts of objects by non-negative matrix factorization. Nature 401(6755):788–791. 10.1038/44565 10548103 10.1038/44565

[CR45] Lodha N, Naik SK, Coombes SA, Cauraugh JH (2010) Force control and degree of motor impairments in chronic stroke. Clin Neurophysiol 121(11):1952–1961. 10.1016/j.clinph.2010.04.005 20435515 10.1016/j.clinph.2010.04.005

[CR46] Maistrello L, Rimini D, Cheung VCK, Pregnolato G, Turolla A (2021) Muscle synergies and clinical outcome measures describe different factors of Upper Limb Motor function in Stroke survivors Undergoing Rehabilitation in a virtual reality environment. Sensors 21(23):8002. 10.3390/s21238002 34884003 10.3390/s21238002 PMC8659727

[CR47] Marchis CD, Severini G, Castronovo AM, Schmid M, Conforto S (2015) Intermuscular coherence contributions in synergistic muscles during pedaling. Exp Brain Res 233(6):1907–1919. 10.1007/s00221-015-4262-4 25821181 10.1007/s00221-015-4262-4

[CR48] McCrea PH, Eng JJ, Hodgson AJ (2005) Saturated muscle activation contributes to Compensatory reaching strategies after stroke. J Neurophysiol 94(5):2999–3008. 10.1152/jn.00732.2004 16014786 10.1152/jn.00732.2004 PMC3471982

[CR49] McMorland AJC, Runnalls KD, Byblow WD (2015) A neuroanatomical Framework for Upper Limb synergies after Stroke. Front Hum Neurosci 9. 10.3389/fnhum.2015.00082 10.3389/fnhum.2015.00082 PMC4329797 25762917

[CR50] Mima Tatsuya T, Keiichiro K, Hallett Mark (2001) Coherence between cortical and muscular activities after subcortical stroke. Stroke 32(11):2597–2601. 10.1161/hs1101.098764 11692023 10.1161/hs1101.098764

[CR51] Moritz CT, Barry BK, Pascoe MA, Enoka RM (2005) Discharge rate variability influences the variation in Force fluctuations across the Working Range of a hand muscle. J Neurophysiol 93(5):2449–2459. 10.1152/jn.01122.2004 15615827 10.1152/jn.01122.2004

[CR52] Muceli S, Boye AT, d’Avella A, Farina D (2010) Identifying Representative Synergy matrices for describing muscular activation patterns during multidirectional reaching in the horizontal plane. J Neurophysiol 103(3):1532–1542. 10.1152/jn.00559.2009 20071634 10.1152/jn.00559.2009

[CR53] Nancy E, Mayo SW-D, Ahmed S, Gordon C, Higgins J, Mcewen S, Salbach N (1999) Disablement following stroke. Disabil Rehabil 21(5–6):258–268. 10.1080/096382899297684 10381238 10.1080/096382899297684

[CR54] Nazarpour K, Barnard A, Jackson A (2012) Flexible cortical control of Task-Specific muscle synergies. J Neurosci 32(36):12349–12360. 10.1523/JNEUROSCI.5481-11.2012 22956825 10.1523/JNEUROSCI.5481-11.2012 PMC3461400

[CR55] Nielsen JB, Brittain J-S, Halliday DM, Marchand-Pauvert V, Mazevet D, Conway BA (2008) Reduction of common motoneuronal drive on the affected side during walking in hemiplegic stroke patients. Clin Neurophysiol 119(12):2813–2818. 10.1016/j.clinph.2008.07.283 18848803 10.1016/j.clinph.2008.07.283

[CR56] Oldfield RC (1971) The assessment and analysis of handedness: the Edinburgh inventory. Neuropsychologia 9(1):97–113. 10.1016/0028-3932(71)90067-4 5146491 10.1016/0028-3932(71)90067-4

[CR57] Ortega-Auriol PA, Besier TF, Byblow WD, McMorland AJC (2018) Fatigue influences the recruitment, but not structure, of muscle synergies. Front Hum Neurosci 12. 10.3389/fnhum.2018.00217 10.3389/fnhum.2018.00217 PMC6021531 29977197

[CR58] Ortega-Auriol P, Byblow WD, Besier T, McMorland AJC (2023) Muscle synergies are associated with intermuscular coherence and cortico-synergy coherence in an isometric upper limb task. Exp Brain Res 241(11):2627–2643. 10.1007/s00221-023-06706-6 37737925 10.1007/s00221-023-06706-6 PMC10635925

[CR59] Pan B, Sun Y, Xie B, Huang Z, Wu J, Hou J, Liu Y, Huang Z, Zhang Z (2018) Alterations of muscle synergies during Voluntary Arm reaching Movement in Subacute Stroke survivors at different levels of impairment. Front Comput Neurosci 12. 10.3389/fncom.2018.00069 10.3389/fncom.2018.00069 PMC6111238 30186130

[CR60] Pizzamiglio S, De Lillo M, Naeem U, Abdalla H, Turner DL (2017) High-frequency intermuscular coherence between arm muscles during Robot-mediated motor adaptation. Front Physiol 7. 10.3389/fphys.2016.00668 10.3389/fphys.2016.00668 PMC5220015 28119620

[CR61] Reyes A, Laine CM, Kutch JJ, Valero-Cuevas FJ (2017) Beta Band Corticomuscular Drive reflects muscle coordination strategies. Front Comput Neurosci 11. 10.3389/fncom.2017.00017 10.3389/fncom.2017.00017 PMC5378725 28420975

[CR63] Roh J, Rymer WZ, Perreault EJ, Yoo SB, Beer RF (2013) Alterations in upper limb muscle synergy structure in chronic stroke survivors. J Neurophysiol 109(3):768–781. 10.1152/jn.00670.2012 23155178 10.1152/jn.00670.2012 PMC3567389

[CR62] Roh J, Rymer WZ, Beer RF (2015) Evidence for altered upper extremity muscle synergies in chronic stroke survivors with mild and moderate impairment. Front Hum Neurosci 9. 10.3389/fnhum.2015.00006 10.3389/fnhum.2015.00006 PMC4324145 25717296

[CR64] Rosenthal R (1986) Meta-Analytic procedures for Social Science Research Sage Publications: Beverly Hills, 1984, 148 pp. Educational Researcher 15(8):18–20

[CR65] Runnalls KD, Ortega-Auriol P, McMorland AJC, Anson G, Byblow WD (2019) Effects of arm weight support on neuromuscular activation during reaching in chronic stroke patients. Exp Brain Res. 10.1007/s00221-019-05687-9 31728596 10.1007/s00221-019-05687-9

[CR66] Safavynia SA, Torres-Oviedo G, Ting LH (2011) Muscle synergies: implications for Clinical Evaluation and Rehabilitation of Movement. Top Spinal Cord Injury Rehabilitation 17(1):16–24. 10.1310/sci1701-16 10.1310/sci1701-16 PMC3143193 21796239

[CR67] Santuz A, Ekizos A, Janshen L, Baltzopoulos V, Arampatzis A (2017) On the methodological implications of extracting muscle synergies from human locomotion. Int J Neural Syst 27(05):1750007. 10.1142/S0129065717500071 27873551 10.1142/S0129065717500071

[CR68] Scano A, Chiavenna A, Malosio M, Molinari Tosatti L, Molteni F (2017) Muscle synergies-based characterization and clustering of Poststroke patients in reaching movements. Front Bioeng Biotechnol 5. 10.3389/fbioe.2017.00062 10.3389/fbioe.2017.00062 PMC5645509 29082227

[CR69] Sosnoff JJ, Newell KM (2006) Are age-related increases in force variability due to decrements in strength? Exp Brain Res 174(1):86. 10.1007/s00221-006-0422-x 16575579 10.1007/s00221-006-0422-x

[CR70] Steele KM, Tresch MC, Perreault EJ (2013) The number and choice of muscles impact the results of muscle synergy analyses. *Frontiers in Computational Neuroscience*, *7*. 10.3389/fncom.2013.00105 10.3389/fncom.2013.00105 PMC3737463 23964232

[CR71] Stinear CM (2017) Prediction of motor recovery after stroke: advances in biomarkers. Lancet Neurol 16(10):826–836. 10.1016/S1474-4422(17)30283-1 28920888 10.1016/S1474-4422(17)30283-1

[CR72] Taylor AM, Christou EA, Enoka RM (2003) Multiple features of motor-unit activity influence force fluctuations during isometric contractions. J Neurophysiol 90(2):1350–1361. 10.1152/jn.00056.2003 12702706 10.1152/jn.00056.2003

[CR73] Tresch MC, Cheung VCK, d’Avella A (2006) Matrix factorization algorithms for the identification of muscle synergies: evaluation on simulated and experimental data sets. J Neurophysiol 95(4):2199–2212. 10.1152/jn.00222.2005 16394079 10.1152/jn.00222.2005

[CR74] Tropea P, Monaco V, Coscia M, Posteraro F, Micera S (2013) Effects of early and intensive neuro-rehabilitative treatment on muscle synergies in acute post-stroke patients: a pilot study. J Neuroeng Rehabil 10(1):1–15. 10.1186/1743-0003-10-103 24093623 10.1186/1743-0003-10-103 PMC3850948

[CR75] Twitchell TE (1951) The restoration of motor function following hemiplegia in man. Brain 74(4):443–480. 10.1093/brain/74.4.443 14895765 10.1093/brain/74.4.443

[CR77] Ward NS (2004) Functional reorganization of the cerebral motor system after stroke. Curr Opin Neurol 17(6):725–730 15542982 10.1097/00019052-200412000-00013

[CR78] Ward NS, Newton JM, Swayne OBC, Lee L, Thompson AJ, Greenwood RJ, Rothwell JC, Frackowiak RSJ (2006) Motor system activation after subcortical stroke depends on corticospinal system integrity. Brain 129(Pt 3):809–819. 10.1093/brain/awl002 16421171 10.1093/brain/awl002 PMC3717515

[CR76] Ward NJ, Farmer SF, Berthouze L, Halliday DM (2013) Rectification of EMG in low force contractions improves detection of motor unit coherence in the beta-frequency band. J Neurophysiol 110(8):1744–1750. 10.1152/jn.00296.2013 23904490 10.1152/jn.00296.2013 PMC3798945

[CR79] Yao J, Chen A, Carmona C, Dewald JPA (2009) Cortical overlap of joint representations contributes to the loss of independent joint control following stroke. NeuroImage 45(2):490–499. 10.1016/j.neuroimage.2008.12.002 19135153 10.1016/j.neuroimage.2008.12.002 PMC2865158

[CR80] Zaaimi B, Edgley SA, Soteropoulos DS, Baker SN (2012) Changes in descending motor pathway connectivity after corticospinal tract lesion in macaque monkey. Brain 135(7):2277–2289. 10.1093/brain/aws115 22581799 10.1093/brain/aws115 PMC3381720

[CR81] Zheng Y, Peng Y, Xu G, Li L, Wang J (2018) Using Corticomuscular coherence to reflect function recovery of Paretic Upper Limb after Stroke: a Case Study. Front Neurol 8. 10.3389/fneur.2017.00728 10.3389/fneur.2017.00728 PMC5767581 29375467

